# A Novel Electrochemical Sensor Modified with a Computer-Simulative Magnetic Ion-Imprinted Membrane for Identification of Uranyl Ion

**DOI:** 10.3390/s22124410

**Published:** 2022-06-10

**Authors:** Li-Qiong He, Zhi-Mei Wang, Yu-Jie Li, Jing Yang, Li-Fu Liao, Xi-Lin Xiao, Yong Liu

**Affiliations:** 1 Hunan Key Laboratory of Typical Environmental Pollution and Health Hazards, School of Public Health, Hengyang Medical School, University of South China, Hengyang 421001, China; 20202014110972@stu.usc.edu.cn; 2 School of Resource & Environment and Safety Engineering, University of South China, Hengyang 421001, China; 17872110128@163.com (Z.-M.W.); 20192002210069@stu.usc.edu.cn (Y.-J.L.); 3 Hengyang Market Supervision Inspection and Testing Center, Hengyang 421001, China; yjawb11@163.com; 4 School of Chemistry and Chemical Engineering, University of South China, Hengyang 421001, China; llfcxllc@163.com; 5 State Key Laboratory of Chemo & Biosensing and Chemometrics, Hunan University, Changsha 410082, China

**Keywords:** computational simulation, magnetic ion imprinting membrane, uranyl ions, electrochemical sensors

## Abstract

In this paper, a novel ion-imprinted electrochemical sensor modified with magnetic nanomaterial Fe_3_O_4_@SiO_2_ was established for the high sensitivity and selectivity determination of UO_2_^2+^ in the environment. Density functional theory (DFT) was employed to investigate the interaction between templates and binding ligands to screen out suitable functional binding ligand for the reasonable design of the ion imprinted sensors. The MIIP/MCPE (magnetic ion imprinted membrane/magnetic carbon paste electrode) modified with Fe_3_O_4_@SiO_2_ exhibited a strong response current and high sensitivity toward uranyl ion comparison with the bare carbon paste electrodes. Meanwhile, the MCPE was fabricated simultaneously under the action of strong magnetic adsorption, and the ion imprinted membrane can be adsorbed stably on the electrode surface, handling the problem that the imprinted membrane was easy to fall off during the process of experimental determination and elution. Based on the uranyl ion imprinting network, differential pulse voltammetry (DPV) was adopted for the detection technology to realize the electrochemical reduction of uranyl ions, which improved the selectivity of the sensor. Thereafter, uranyl ions were detected in the linear concentration range of 1.0 × 10^−9^ mol L^−1^ to 2.0 × 10^−7^ mol L^−1^, with the detection and quantification limit of 1.08 × 10^−9^ and 3.23 × 10^−10^ mol L^−1^, respectively. In addition, the sensor was successfully demonstrated for the determination of uranyl ions in uranium tailings soil samples and water samples with a recovery of 95% to 104%.

## 1. Introduction

Uranium and its compounds can be utilized as fuel for nuclear power plants, for the production of tanks, armor, and armor-piercing ammunition, for the coloring of ceramic products, and for the electron microscopy studies of biological samples [1]. As these applications may be harmful to mankind’s health or the environment, it is essential to rapidly and accurately detect trace uranyl ions in environmental, geochemical, or clinical samples [2]. Uranium has different valence states, mainly in the form of two valence states of U^4+^ and U^6+^ and other metal compounds or oxides. The latter is easy to form water-soluble uranyl ion (UO_2_^2+^) compounds. Uranium in water samples has been determined using various physicochemical methods, including inductively coupled plasma mass spectrometry (ICP-MS) [3], in some cases combined with ion chromatography [4] and radiation measurement techniques [5,6], and other analytical methods such as differential pulse polarography [7], neutron activation analysis [8], gas chromatography [9,10], as well as γ and α spectrum [11]. Although these methods have high sensitivity and favorable detection limits, their application requires costly equipment and lofty operating expenses. Furthermore, some methods even demand preliminary separation steps such as extraction and ion exchange procedures [12] for sample preparation. Comparatively, electroanalytical techniques [13] are relatively effective because they are simple to operate, low in cost, and can reach extremely low detection limits. Commonly used electrochemical analysis techniques are adsorptive stripping voltammetry (AdSV) [14,15,16], differential pulse voltammetry (DPV) [17] and cyclic voltammetry (CV) [18]. Due to its portability and low power requirements, voltammetry technology is particularly suitable for on-site monitoring of uranium [19,20]. Differential pulse voltammetry is considered to be a powerful method for the determination of trace metals, allowing the simultaneous measurement of multiple elements with high sensitivity [21,22].

Molecular imprinting technology (MIT), also known as molecular template technology, is a process that combines molecular recognition and specificity to prepare novel polymers with selective recognition ability for specific target molecules (template molecules, imprinted molecules). Molecular imprinting technology is widely used in the preparation of chemically modified electrodes and electrochemical sensing systems for its advantages of good stability, low cost, and ease of preparation. However, the optimal conditions for the synthesis of molecularly imprinted polymers are determined by extensive experiments. In recent years, with the fast and rapid development of polymer materials chemistry, computer simulations have been widely applied to the study of molecularly imprinted systems. The use of computers for molecular simulations mainly includes two aspects, namely, computational chemistry and molecular simulations. Computational chemistry refers to the application of computational methods to chemistry to solve practical problems, including ab initio algorithms based on simple molecules and kinetic calculations of complex molecules, while computer molecular simulation refers to the general method of constructing actual atomic models to describe large and complex chemical systems and to predict their macroscopic physical properties. When the spatial position of each nucleus in the computational system is determined, the distribution of electron density in space can be determined, and then the energy of the computational system can be represented by a generalized function of electron density. For the computational system, a suitable density generalization method can yield a more accurate solution than the ab initio algorithm, and at the same time take less time. Commonly used density generalization methods include B3LYP, etc. Due to the limitation of computational resources, the more common 6-31G(d) all-electron basis group is used for light atoms, while the Lanl2DZ pseudopotential basis group is used for transition metal atoms in order to eliminate relativistic effects. Finally, a more reasonable calculation method was chosen by comparing the errors of the calculated and experimental values of each complex structure. With the development of computer technology and quantum chemistry research theories, computer simulations have been applied to molecular imprinting preparation [23]. Liu et al. [24] used chloramphenicol (CAP) as a template molecule and methacrylic acid (MAA) as a functional binding ligand. All calculations were performed using Gaussian 09 software at the LC-WPBE/6-31g(d, p) level using density generalized function theory [25]. The conformations of the complexes formed by CAP and MAA in different ratios were optimized and the solvation energies of the complexes in the above-mentioned solvents were discussed to check the optimal geometrical conformation and the strength of the interaction between these solvents. Nanomicrospheres prepared by precipitation polymerization can be used as sorbent materials in extraction columns due to their uniform particle size and microporous structure [26]. Guided by the calculation results, molecularly imprinted polymers were synthesized by precipitation polymerization and the adsorption capacity of molecularly imprinted polymers was investigated by equilibrium adsorption experiments. In recent years, more and more researchers have made great progress in order to explore the molecular imprinting process and molecular recognition mechanism, reducing the blindness of preparation, improving the efficiency of molecularly imprinted polymers development, and increasing the adsorption, selectivity, and stability of molecularly imprinted polymers. Many studies have used B3LYP, M062X, and PBE0 methods to simulate and design molecular imprinting systems by using Gaussian software. Zhao et al. [27] used formaldehyde (HCHO) as the imprinting molecule, MAA as the functional binding ligand, divinylbenzene, ethylene glycol dimethacrylate (EGDMA), pentaerythritol triacrylate and trimethylolpropane as crosslinkers, and water as the solvent to construct the system. Under the condition that the molecular structures of the imprinted molecules and the functional binding ligands of MAA were modeled, the structures of HCHO and MAA were optimized using quantum chemical density flooding theory. After selecting the most stable conformation, the most suitable crosslinker and solvent were selected by calculating the binding energy and hydrogen bond number.

Ion imprinted polymers (IIPs) are highly selective synthetic receptors that can recognize metal ions, which also retains all the advantages of molecularly imprinted polymers (MIPs) in this work. The formation of the polymer exhibits the selective binding of specific cations, including the formation of coordination complex agent cavities, the arrangement of which matches the charge, coordination number, coordination geometry, and size of the target cation. Although the bulk ion-imprinted polymer prepared by the traditional method has high selectivity [28,29] it also has drawbacks such as uneven distribution of binding sites, embedding of most of the binding sites, and poor accessibility of the template molecule sites [30]. Hence, research has shifted towards attaining highly uniform spherically imprinted particles, especially at the nanometer level [31]. Recently, magnetic nanoparticles (MNP) have attracted considerable attention owing to their distinctive performance and biocompatibility for various applications in magnetic resonance imaging (MRI) [32], biosensors, and biochemical product separation. In modified electrodes, different forms of electrode materials such as conductive polymers [33,34] and MNPs [35] in the sensor show synergistic effects while enhancing the performance of the sensor. This silica-coated MNP can provide an effective platform for electrochemical polymerization (ECP) of the materials. Fe_3_O_4_@SiO_2_ magnetic nanoparticles have the characteristics of stable chemical properties, good dispersibility and water solubility, and strong physical structure. Hence, they are the most common substance in imprinting technology. The methods used to obtain IIPs nanoparticles include suspension, multi-step solubilization and precipitation polymerization [36,37]. Among them, the precipitation technology is one of the most convenient because it is a homogeneous, one-step synthesis that does not require the use of surfactants or stabilizers.

The preparation of MIPs by precipitation polymerization is primarily reported [38]. Compared with the polymer prepared by bulk polymerization [39], the polymer particles prepared by this procedure have a more uniform particle size than those prepared by native polymerization, eliminating the need for steps such as crushing and sieving. It remains challenging to select functional binding ligands, solvents, and other conditions to prepare polymers. The drawback of theoretical guidance leads to the long preparation time and high cost. The use of molecular simulation to rationally calculate the various polymerization conditions required for imprinted polymers, such as the selection of optimal functional binding ligands, reaction solvents, etc., which can improve the success rate of experiments and reduce the waste of resources. Consequently, the development of computer technique and quantum chemistry theory [40,41] is of particular importance for the application of ion imprinting systems.

## 2. Materials and Methods

### 2.1. Reagents 

All chemicals were used for analytical purity. Methacrylic acid (MAA), 2,5-Pyridinedicarboxylic acid (H_2_Pdc), 2,2’-Azobisisobutyronitrile (AIBN), Ethylene glycol dimethacrylate (EGDMA), Dimethyl sulfoxide (DMSO), Ethyl orthosilicate (TEOS), Sodium acetate (NaAc), Ethylene glycol, Ethanol, Ammonia and FeCl_3_·6H_2_O were all purchased from Aladdin Chemical Reagent Co., Ltd. (Shanghai, China). Uranyl (VI) nitrate hexahydrate was obtained from Hubei Chushengwei Chemical Co., Ltd. (Wuhan, China). Distilled water was used in the experiment. 

### 2.2. Apparatus

All electrochemical measurements were carried on the CHI-660C electrochemical workstation (Chenhua Co. Ltd., Shanghai, China). Surface analysis was carried out in a S4800 scanning electron microscope (Hitachi Co., Ltd., Tokyo, Japan) and TALOS F200 transmission electron microscope (FEI Co. Ltd., Waltham, MA, USA), DZF-6020 vacuum drying oven (Shanghai Sanfa Scientific Instrument Co., Ltd., Shanghai, China), pHs-10C digital acidity meter (Shanghai Lei Magnetic Scientific Instrument Factory, Shanghai, China).

### 2.3. Design and Calculation of Magnetic Ion Imprinted Membrane by Molecular Simulation

The theoretical model of functional binding ligand template complexation was devised and the application of IIP in computational simulation is discussed. The selection of functional binding ligands is critical to the successful synthesis of IIPs with excellent properties. The complex model was established by Gaussian view program, and the B3LYP density functional theory was employed to optimize the configuration of UO_2_^2+^ and functional binding ligands under the 6-31+G group to calculate the binding energy (Δ*E*) for the formation of 1:2 configuration between the template ion and the binding ligand [42], which was calculated as follows:

Δ*E* = *E*_C_ − *E*_T_ − ∑*E*_M_

where Δ*E* is the difference in binding energy; *E_C_* is the total energy of the binding ligand template structure; *E_T_* is the energy of UO_2_^2+^; Σ*E_M_* is the sum of the energy of the functional binding ligands.

### 2.4. Fabrication of Ion Imprinted Membrane

#### 2.4.1. Synthesis of Fe_3_O_4_@SiO_2_ Nanoparticles

During the synthesis of Fe_3_O_4_ nanoparticles, 2.7 g FeCl_3_·6H_2_O and 7.2 g NaAc were dissolved in a 200 mL beaker with 80 mL of ethylene glycol solution and stirred by ultrasonic 30 min until they were completely dissolved. The oxygen of the sample solution was dislodged by bubbling nitrogen through the sample for 10.0 min and transferred to a high-pressure reaction kettle, heated at 200 °C for 8h to produce Fe_3_O_4_ magnetic nanoparticles. The obtained Fe_3_O_4_ nanoparticles were simply separated by external magnetic forces and the supernatant was leached out. Subsequently, it was cleaned with distilled water and ethanol for several times to reach pH = 7.0, and then dried in a vacuum drying oven. Prior to the silica coating process, 1.0 g of the black precipitate of Fe_3_O_4_ was ultrasonic treated at room temperature for 1 h and dispersed in a 120 mL of ethanol solution. A total of 5.0 mL NH_3_·H_2_O and 5.0 mL TEOS were added dropwise to form silica-coated MNP in a beaker and stirred at room temperature for 6 h. The brown-yellow solid was separated by magnetic separation and washed with water and ethanol for several times until it was neutral, then dried at 60 °C for 8 h to collect Fe_3_O_4_@SiO_2_ particles with a particle size of 55–75 nm.

#### 2.4.2. Preparation of Carbon Paste Electrodes Modified with Fe_3_O_4_@SiO_2_

The 4.0 g of pure graphite powder, 1 mL of paraffin oil and ethanol were added as the solvents in a 25 mL beaker, which were continuously stirred into a uniform paste, followed by ultrasonic treatment for 20 min. The mixture was then dried in an oven at 60 °C for 6 h to evaporate the solvent. The graphite block was ground into the powder with a glass rod, and then the powder was filled into a polyethylene plastic pipe with a diameter of 3.5 mm and a length of 5 cm. A round rubidium iron boron magnet (4.0 mm in diameter and 2.0 mm in thickness) was inserted about 1.5 mm near the mouth of the tube, then the prepared paste powder was filled until it was level with the mouth of the tube, and the other end was inserted into a pencil core with a length of about 6.0 cm. Finally, one end of the filler magnet was polished on sandpaper to make its mirror and the electrode polished on smooth paper to obtain a smooth surface of the electrode.

#### 2.4.3. Preparation of Imprinted Polymer

**Figure 1:**
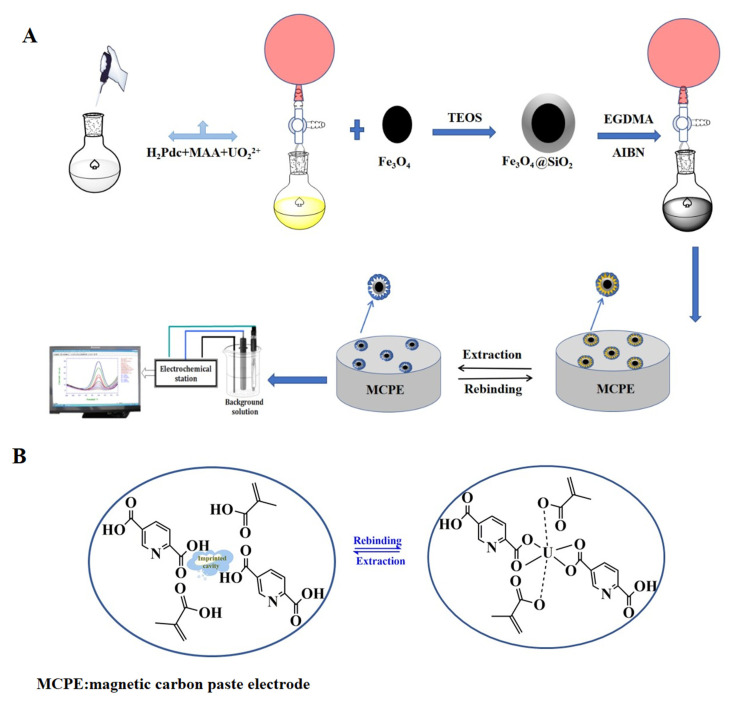
Diagrammatic sketch of (**A**) the preparation of MIIP/Fe_3_O_4_@SiO_2_/MCPE sensor; (**B**) the MIIP/Fe_3_O_4_@SiO_2_/MCPE sensor template extraction-rebinding mechanism.

In the current experiment, the nano-UO_2_^2+^ imprinted polymer was fabricated by precipitation polymerization. In the first step, 2 mmol of methacrylic acid (MAA) and 2 mmol of pyridine-2,5-dicarboxylic acid (uranyl-binding ligand) were dissolved in 10 mL of DMSO and placed in a 100 mL round bottom flask. In the second step, 1 mmol UO_2_(NO_3_)_2_ of imprinted metal ion (template) was slowly added to the round bottom flask and stirred for 2 h at room temperature. In the third step, 100 μL EGDMA and 0.05g AIBN were added as crosslinking agent and initiator. In addition, 0.05 g Fe_3_O_4_@SiO_2_ was also further augmented. The oxygen was removed from the sample solution by agitation of nitrogen in the sample for 10 min. The polymerization was conducted in an oil bath at 65 °C with magnetic stirring at 300 rpm for 12 h. To remove the unreacted material, the prepared polymer was washed with 1:4 (*v*/*v*) methanol/water for several times. Then, the imprinted metal ions (uranyl ions) were leached out with HCl (0.5 mol L^−1^). Until there was no uranyl ion in the washing solution. Comparing the potential value of the eluted electrode with the blank group, it can be assumed that there is no uranyl ion in the solution. Eventually, it was cleaned with double distilled water until the pH value is neutral, dried in vacuum at 60 °C for 8 h, and then set aside. Figure 1A presents the experimental process.

The non-ionic imprinted polymer was prepared by the same way without adding template ion (UO_2_^2+^). By means of the effect of a magnetic field, the magnetically imprinted polymer can be firmly fixed on the electrode surface. The non-ionic imprinted polymer was arranged by the same way without template ion (UO_2_^2+^). Then, 1.0 mg of the above fabricated polymer was sonicated and dispersed in 1.0 mL of tetrahydrofuran, and 25 μL of the suspension was uniformly dropped onto the surface of the MCPE to obtain the sensor. The particular mechanism is demonstrated in Figure 1B.

### 2.5. Electrochemical Measurements

The determination platform of this experiment was performed the CHI-660C electrochemical workstation. The carbon paste electrode modified by the ion imprinted membrane was used as the working electrode, the calomel electrode was used as the reference electrode, and the platinum electrode was used as the counter electrode. Electrochemical experiments were implemented on the basis of the optimized conditions. Prior to the experiment, the working electrode was recombined in 5.0 mL UO_2_^2+^ standard solution for 20 min, followed by washing with doubly distilled water to remove the surface impurities and immersed in 5.0 mL 1 mol L^−1^ (pH = 6.0) 2-morpholino-ethanesulfonic acid (MES) buffer solution and 0.1 mol L^−1^ KCl supporting electrolyte. Then, DPV was executed in the potential range from 0.0 V to −0.5 V with a potential increment of 20 mV, the pulse width of 170 ms, the pulse amplitude of 50 mV and the scanning rate of 25 mV s^−1^.

Electrochemical impedance spectroscopy (EIS) was employed to characterize the charge transfer characteristics and electrochemical performance of different modified sensors, which were gauged using 1.0 mmol L^−1^ K_3_Fe(CN)_6_/K_4_Fe(CN)_6_ (1:1) as an electroactive probe in 0.1 mol L^−1^ KCl electrolyte with a bias potential of 0.25 V and a frequency range of 0.1–10^5^ Hz with a signal amplitude of 5 mV.

The influence of the supporting electrolyte such as HAc-NaAc, sodium citrate, Tris-HCl, MES(2-(N-Morpholino) ethanesulfonic acid hydrate) and phosphate acid-sodium phosphate on the peak current intensity of the sensor surface was investigated. The effect of pH value on the electrochemical determination of 1.5 × 10^−7^ mol L^−1^ UO_2_^2+^ solution was studied in the range of 4.0–9.0. To that end, the prepared electrodes were inserted into the solutions with different pH values where they were incubated for 10 min at a constant stirring speed and then measured.

Repeatability: Six tests were performed using the same electrodes MIIP/Fe_3_O_4_@SiO_2_/MCPE for 0.2, 0.15, and 0.1 μmol L^−1^ UO_2_^2+^ solutions, respectively; the reproducibility of the sensors was evaluated by measuring the response signals of the electrochemistry of the six sensors in the above uranyl solutions under the same time conditions.

Stability: The prepared sensors were stored at dry room temperature for 30 days and the signals were detected at five-day intervals.

### 2.6. Evaluation of the Electroactive Surface Area of the Electrodes

The electroactive surface areas of the Fe_3_O_4_@SiO_2_/MCPE, MIIP/Fe_3_O_4_@SiO_2_/MCPE, MIIP/MCPE, N-MIIP/MCPE was evaluated by cyclic voltammetry at different scanning rates between 0 V and −0.5 V, 1.0 mmol L^−1^ K_3_Fe (CN)_6_ was used as the redox probe and 0.1 mol L^−1^ KCl as the electrolyte. The reversible process was carried out at room temperature (298.15 ± 2 K) and the electroactive surface area could be calculated by the Randles-Sevcik formula [43]:

*I_p_* = (2.69 × 10^5^) *n*^3/2^*AD*^1/2^*C*_0_*v*^1/2^

*I_p_* is peak current (A), *n* is the number of electron transfers, *A* is the electrode surface area (cm^2^), *D* is the diffusion coefficient (cm^2^ s^−1^), *C*_0_ is the concentration of the probe (mol cm^−3^), *v* is the scanning rate (V s^−1^). The electrochemical reduction behavior of Fe (CN)_6_^3−^ to Fe (CN)_6_^4−^ can be reflected by plotting these peak current amplitudes. When plotting the square root of the scanning rate, a linear fitting with a slope of (2.69 × 10^5^) *n*^3/2^*AD*^1/2^*C*_0_ can be obtained. For 1.0 mmol L^−1^ K_3_Fe (CN)_6_ in 0.1 mol L^−1^ KCl electrolyte, *n* = 1, *D* = 7.6 × 10^−6^ cm^2^ s^−^^1^.

### 2.7. Actual Sample Analysis

In order to attest that the designed MIIP/Fe_3_O_4_@SiO_2_/MCPE sensor is applicable to different environmental analysis, the samples taken for this experiment were from three different soils in the area surrounding the uranium tailings in Hunan and water samples were from the Xiang jiang River basin, as well as the tap water used in daily use. The water samples were simply treated with filter paper to remove floating impurities, and soil samples were acidified by hydrochloric acid, nitric acid, hydrofluoric acid and perchloric acid in small amounts several times to form the solutions. The above samples were assayed and recovered by the standard addition method with six tests performed in parallel for each sample through the process. In the appropriate linear range, 0.30 μmol L^−1^ and 0.50 μmol L^−1^ of UO_2_^2+^ solutions were added to soil and water samples to be tested.

### 2.8. Statistical Treatment of the Data

The detection and quantification limits were calculated as 3S/K and 10S/K, respectively, where S was the standard deviation of the calibration graph intercept and K—the calibration graph slope.

## 3. Results and Discussion

### 3.1. Simulation Design of Ion Imprinted Polymer

**Figure 2:**
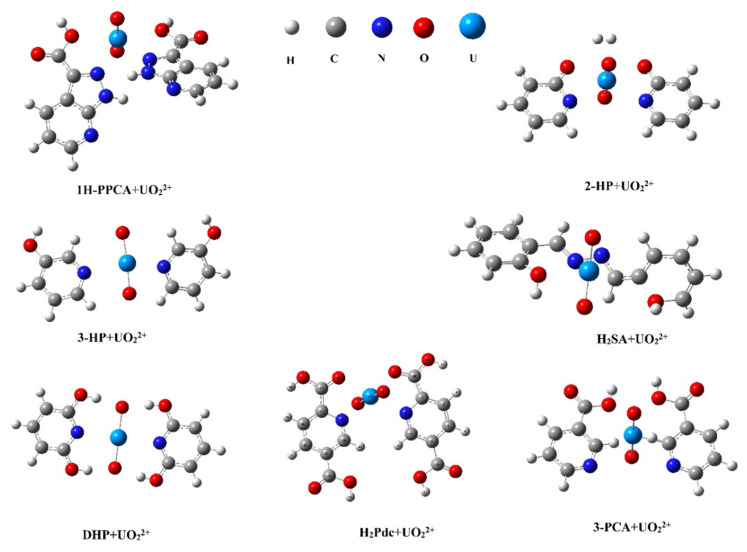
Optimized conformation of UO_2_^2+^ and functional binding ligand.

Screening for suitable functional binding ligands remains a key factor in the design of the template binding ligand model. Therefore, some functional binding ligands that may have strong affinity to uranyl ions were selected in this study. Based on computational chemistry methods, several functional binding ligands such as 1H-pyrazolo[3,4-B] pyridine-3-carboxylic acid (1H-PPCA), 2-hydroxypyridine (2-HP), 3-hydroxypyridine (3-HP), salicylaldehyde acridine (H_2_SA), 2,6-dihydroxypyridine (DHP), pyridine-2,5-dicarboxylic acid (H_2_Pdc), and nicotinic acid (3-PCA) were screened. The structures of 1H-PPCA [44], 3-PCA [45], H_2_SA [46], and H_2_Pdc [47] were chosen as binding ligands because of their strong interaction with uranyl ions; 2-HP, 3-HP, and D-HP may have some affinity with uranyl ions because they contain pyridine and hydroxyl structures [48]. As presented in Figure 2, the structure of individual molecules was first optimized by density functional methods (Figure S1), and then the configuration of the complexes was optimized structurally after binding of functional binding ligands to template ions. The binding energies of the optimized template functional binding ligands (1H-PPCA, 2-HP, 3-HP, H_2_SA, DHP, H_2_Pdc, 3-PCA) complexes were computed, as seen in Table 1. Despite the strong interaction of the 1H-PPCA and 3-PCA structures with UO_2_^2+^ [49,50], there is still a considerable spatial potential barrier with the template ion, leading to the low binding energy. In summary, the system composed of pyridine-2,5-dicarboxylic acid +UO_2_^2+^ was the best complex system for the experiment, as seen in Figure 2. Since the simple structure of pyridine-2,5-dicarboxylic acid is more readily associated with UO_2_^2+^ due to its lower spatial potential resistance when it binds to the template. Additionally, the structure formed is relatively stable. Therefore, the binding capacity of pyridine-2,5-dicarboxylic acid +UO_2_^2+^ is also maximized.

### 3.2. Electrochemical Characterization of IIP/Fe_3_O_4_@SiO_2_/MCPE Sensors

#### 3.2.1. Differential Pulse Voltammetry (DPV) Characterization

**Figure 3:**
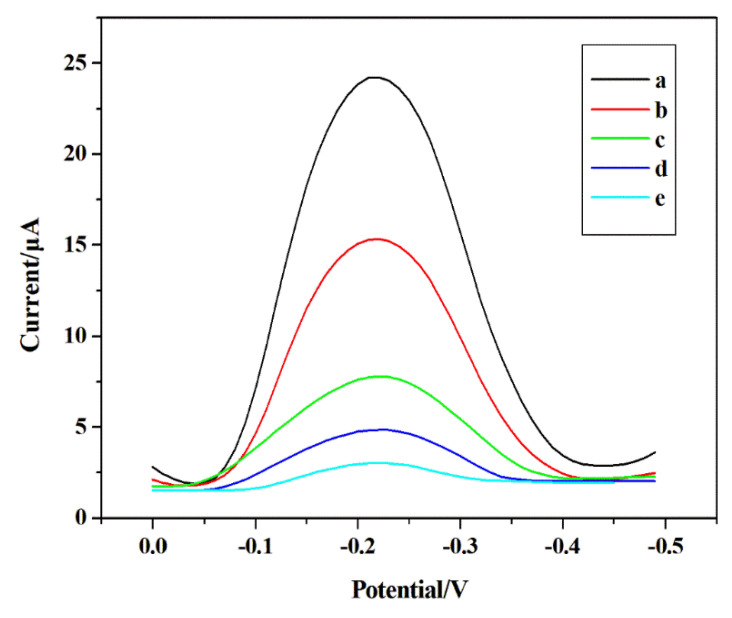
Schematic diagram of response current DPV of (a) Determination of UO_2_^2+^ after elutionrecombination of MIIP/Fe_3_O_4_@SiO_2_/MCPE in 1.5 × 10^−7^ mol L^−1^ UO_2_^2+^ solution for 20 min, (b) Determination of UO_2_^2+^ by MIIP/Fe_3_O_4_@SiO_2_/MCPE with template, (c) Determination of UO_2_^2+^ by N-MIIP/Fe_3_O_4_@SiO_2_/MCPE, (d) Determination of UO_2_^2+^ by MCPE, (e) Determination of UO_2_^2+^ by MIIP/Fe_3_O_4_@SiO_2_/MCPE after eluting the template.

The electrochemical activity of the electrodes on the different sensors was examined by differential pulse voltammetry (DPV) under optimal conditions (Figure 3). There exists no obvious reduction peak of UO_2_^2+^ on the exposed MCPE (Figure 3d). The smaller reduction peak current at the N-MIIP/Fe_3_O_4_@SiO_2_/MCPE (carbon paste electrode modified by non-ion imprinted membrane) (Figure 3c) indicates that the adsorption of uranyl ions on the electrode surface remains relatively low. However, the clear and strong peak observed at the MIIP/Fe_3_O_4_@SiO_2_/MCPE (Figure 3b) was due to the accumulation of UO_2_^2+^ on the sensor surface. Meanwhile, the removal of UO_2_^2+^ and recombination (Figure 3a) exhibited high currents compared with other electrodes, indicating that the ion-imprinted cavities have superior memory recognition and binding ability for template ions. Besides, it can also be found that the conductive copolymer layer in the IIP manifests significant electrocatalytic effect by accelerating the electron transfer and electroreduction phenomena. The peak current of UO_2_^2+^ at MIIP/Fe_3_O_4_@SiO_2_/MCPE is evidently higher than those at bare MCPE and N-MIIP/Fe_3_O_4_@SiO_2_/MCPE, manifesting that there is a selective cavity and normal operation in the IIP formed during polymerization. Besides, the template-free MIIP/Fe_3_O_4_@SiO_2_/MCPE did not show the signal response after removing the template ions (Figure 3e), demonstrating that UO_2_^2+^ was effectively removed. Moreover, the reduction peak of UO_2_^2+^ appears at −0.22 V on MIIP/Fe_3_O_4_@SiO_2_/MCPE. Compared with the N-MIIP/Fe_3_O_4_@SiO_2_/MCPE, the increase of peak current on the MIIP/Fe_3_O_4_@SiO_2_/MCPE show the electrocatalytic activity of IIP for the reduction mechanism of uranyl ion. According to the obtained results, MIIP/Fe_3_O_4_@SiO_2_/MCPE provides a promising electrochemical sensor for the determination of UO_2_^2+^.

#### 3.2.2. Study on Accessible Surface Area of Different Sensors

The average value of the electroactive surface area of the MIIP/Fe_3_O_4_@SiO_2_/MCPE after removing the template ion is 0.975 ± 0.005 cm^2^, which can be obtained from the slope (2.69 × 10^5^ *n*^3/2^*AD*^1/2^*C*_0_). It is 1.8 times that of the MIIP/Fe_3_O_4_@SiO_2_/MCPE with template ions (0.541 ± 0.003 cm^2^), 1.6 times that of MIIP/MCPE (0.609 ± 0.005 cm^2^), 1.5 times that of N-MIIP/Fe_3_O_4_@SiO_2_/MCPE (0.650 ± 0.003 cm^2^) and 3.2 times that of Fe_3_O_4_@SiO_2_/MCPE (0.305 ± 0.003 cm^2^). It indicates that the effective removal of template UO_2_^2+^ leaves many imprinted holes on the surface of MIIP/Fe_3_O_4_@SiO_2_/MCPE.

#### 3.2.3. Surface Morphological Characterization of Fe_3_O_4_ and Fe_3_O_4_@Sio_2_

By analyzing the morphology of the particles in Figure S2A,B, it is found that Fe_3_O_4_@SiO_2_ has been formed in the particles in Figure S2B, indicating that the surface of Fe_3_O_4_ has been successfully wrapped by SiO_2_ (uniformly) to form a core-shell structure.

#### 3.2.4. Characterization of Sensor Surface Morphology

**Figure 4:**
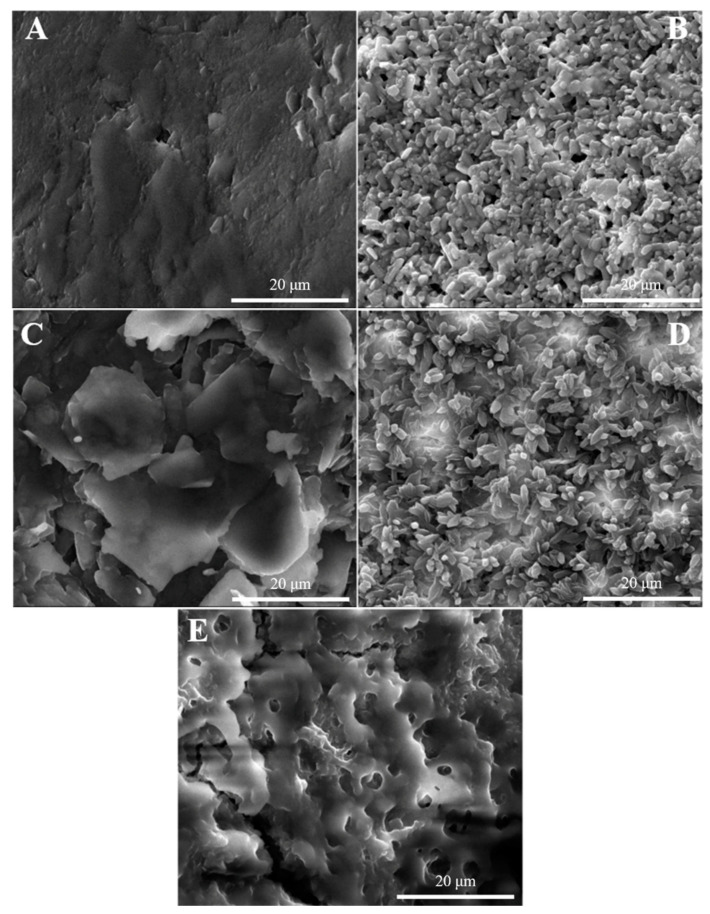
The SEM images of (**A**) MCPE, (**B**) MIIP/MCPE, (**C**) N-MIIP/Fe_3_O_4_@SiO_2_/MCPE, (**D**) MIIP/Fe_3_O_4_@SiO_2_/MCPE with template, (**E**) MIIP/Fe_3_O_4_@SiO_2_/MCPE after removal of the template.

Scanning electron microscopy (SEM) images characterize the surface roughness and micromorphology on the nanoscale in order to better study the elution and adsorption processes of uranyl ions. Figure 4A–E shows the surface topography of bare MCPE, MIIP/MCPE, N-MIIP/Fe_3_O_4_@SiO_2_/MCPE, MIIP/Fe_3_O_4_@SiO_2_/MCPE before elution, and MIIP/Fe_3_O_4_@SiO_2_/MCPE after elution utilizing SEM. As can be seen from Figure 4A, the surface of the electrode was relatively flat and there is basically no polymer formation. While MIIP/MCPE can be observed that lots of spherical particles were deposited on the surface of the imprint electrode (Figure 4B), the IIP tube diameter with Fe_3_O_4_@SiO_2_ modification attached was larger compared to the Figure 4B modified electrode, which also indicates that the larger specific surface area was conducive to IIP attachment and better dispersion with smaller particle size (Figure 4D). However, the surface of the non-ionic imprinted polymer modified electrode was disordered because the surface of electrode N-MIIP/Fe_3_O_4_@SiO_2_/MCPE did not form imprinted cavities (Figure 4C). As presented in Figure 4E, the surface morphology of the sensor became rough and loose after the template ion elution, showing a porous morphology. Meanwhile, numerous tiny ‘imprinted holes’ can be observed. This is due to the fact that the template ion went away the polymer membrane, an imprinted pore matching the structure of the template ion would be left on the polymer membrane. Depending on the shape of the imprinted pore and the site of recognition, the imprinted polymer membrane can be recognized specifically and reabsorbed. The existence of these template ions ‘imprinted holes’ contributes a lot to improving the adsorption selectivity.

#### 3.2.5. Electrochemical Impedance Diagram

The results of EIS were represented by Nyquist plots (Figure S3) and analyzed using the Randles equivalent circuit (inset of Figure S3), where Z′ and Z″ were the real and imaginary parts of the impedance, respectively. As presented in Figure S3, the electrochemical impedance data were fitted using Zview software to acquire the R_ct_ values for the different electrodes. The charge transfer resistance (R_ct_) value of the bare MCPE was 2207 Ω (Figure S3e). When the bare MCPE was coated with MIIP/Fe_3_O_4_@SiO_2_, the R_ct_ value decreased to 1684 Ω (Figure S3d), indicating that the successful construction of the conductive copolymer layer promotes charge transfer, which is due to the acceleration of the charge transfer process by the magnetic field [51]. After the formation of the IIP ion imprinted membrane, the ion imprinted membrane was formed by hydrolysis and condensation under the condition where UO_2_^2+^ serves as a template in the presence of binding ligand and crosslinker, and then removed the UO_2_^2+^. The R_ct_ value is correspondingly reduced from 1684 Ω (MIIP/Fe_3_O_4_@SiO_2_/MCPE with template) (Figure S3d) to 518 Ω (MIIP/Fe_3_O_4_@SiO_2_/MCPE without template) (Figure S3c), objectively revealing the successful construction of the MIIP/Fe_3_O_4_@SiO_2_/MCPE channel. When the MIIP/Fe_3_O_4_@SiO_2_/MCPE with template ion removed were immersed in 1.5 × 10^−7^ mol L^−1^ UO_2_^2+^ solution, the imprinting holes were occupied by the template ions. Therefore, the R_ct_ value greatly increases significantly from 518 Ω to 905 Ω (Figure S3b), meaning that some of the channels through which the redox probe passes were blocked. It turned out as expected, the N-MIIP/Fe_3_O_4_@SiO_2_/MCPE R_ct_ value of 2510 Ω (Figure S3f) was much larger than the R_ct_ value of the removal of template ions since the N-MIIP/Fe_3_O_4_@SiO_2_/MCPE lacks such channels for the probe to pass through. The difference in R_ct_ values between MIIP/Fe_3_O_4_@SiO_2_/MCPE and MIIP/MCPE (775 Ω, Figure S3a) revealed that the Fe_3_O_4_@SiO_2_ of the modified electrode can promote the charge transfer of probe Fe (CN)_6_^3−/4−^.

### 3.3. Optimization of Experimental Conditions

#### 3.3.1. Dosage Optimization

In the process of preparing imprinted polymers by precipitation polymerization, the ligand H_2_Pdc complexes with uranyl ion and functional binding ligand MAA to form the polymer. The selection of the cross-linking agent and its amount, the amount of AIBN, and the reaction time of forming an imprinted membrane are also crucial during the experiment. In the synthesis of polymeric materials, the choice of cross-linking agent is also essential. If the force between the crosslinking agent and the template molecule is slight, the stable polymer cannot be shaped. However, if the force is too large, it will cause an excessive reaction between the binding ligand and the template ion, which is difficult to elute. In this experiment, 1,3,5-trimellitic acid (TRIM), ethyl orthosilicate (TEOS), ethylene glycol dimethacrylate (EGDMA), methyl orthosilicate (TMOS), diethylene Benzene (DVB) were investigated for research, and these crosslinking agents were used to prepare different MIIP/MCPE. The electrochemical effect was detected by DPV based on the intensity of the reduction peak current value. From Figure S4, it can be concluded that the current value of the electrochemical sensor prepared by EGDMA is the strongest. That is, EGDMA was selected as the optimal crosslinking agent in this experiment.

The amount of crosslinking agent EGDMA (Figure S5A), the amount of AIBN (Figure S5B), and the reaction time of forming imprinted membrane (Figure S5C) were analyzed by the single factor method. The crosslinking agent could not connect the polymer and template well when EGDMA was lower than 100 μL. If EGDMA was higher than 100 μL, the binding ligand and template would react excessively to form colloid, which would hinder the formation of imprinted sites, thus affecting the sensitivity of the sensor. As revealed by the research, the most desirable performance of the sensor was manufactured with 100 μL of EGDMA, 0.05 g of initiator AIBN, and 12 h of reaction time. The sensitivity of the constructed sensor was the highest and the response value was the best.

#### 3.3.2. Optimization of Eluent and Elution Time

Whether the template elution is clean and the cavity structure on the molecular surface is complete are the pivotal elements that affect the success of the sensor preparation. In order to effectively remove the template ions, the elution efficiency of desorbing UO_2_^2+^ from MIIP/Fe_3_O_4_@SiO_2_/MCPE electrode with inorganic acid eluent was investigated, as shown in Figure S6. The effects of the properties of inorganic acids on the desorption of UO_2_^2+^ were examined by using 5.0 mL of 1 mol L^−1^ HCl, 1 mol L^−1^ HNO_3_, 1 mol L^−1^ H_2_SO_4_ and 1 mol L^−1^ CH_3_COOH. Desorption rate was obtained by measuring the potential values before and after elution. As demonstrated by the experimental results, desorption rates were 98%, 91%, 50%, and 76%, respectively. Therefore, 1 mol L^−1^ HCl was selected as the leaching agent over several other inorganic acids. For the sake of investigating the optimal concentration of the leaching solution, several hydrochloric acid solutions with different concentrations (0.1, 0.2, 0.5, and 1.0 mol L^−1^, respectively) were utilized to extract UO_2_^2+^ ions from imprinted sites of polymer matrix during quantitative desorption. The results showed that the desorption of UO_2_^2+^ ions increased with the increase of hydrochloric acid concentration which is most likely owing to the increased protonation of ligand heteroatoms in the polymer networks. Hence, 0.5 mol L^−1^ was chosen as the optimum eluent in the voltammetry of uranyl ions. As well, the effect of the elution time on the peak current was analyzed, and the optimum elution time was 15 min.

#### 3.3.3. Effect of pH

**Figure 5:**
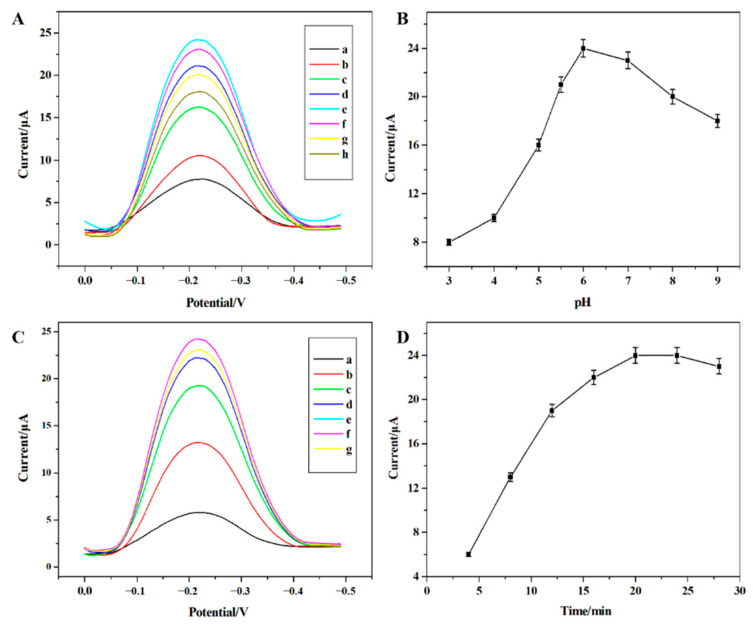
The *voltammograms* of the MIIP/Fe_3_O_4_@SiO _2_/MCPE electrode when detecting of UO_2_^2+^ (**A**,**B**) the effect of pH (a: pH = 3, b: pH = 4, c: pH = 5, d: pH = 5.5, e: pH = 6, f: pH = 7, g: pH = 8, h: pH=9); (**C**,**D**) the effect of enrichment time (a: 4 min, b: 8 min, c: 12 min, d:16 min, e: 20 min, f: 24 min, g: 28 min).

In the experimental process, the pH value of the electrolyte has an important effect on the current intensity of the electrode surface. The influence of the supporting electrolyte such as HAc-NaAc, sodium citrate, Tris-HCl, MES(2-(N-Morpholino) ethanesulfonic acid hydrate), and phosphate acid-sodium phosphate on the peak current intensity of the sensor surface was investigated. As can be perceived from Figure 5A, the electrode response decreases sharply when the pH value is below 6. This may be due to the protonation of nitrogen atoms in the H_2_Pdc functional groups, which weakens the interaction of UO_2_^2+^ with the electron pairs of nitrogen atoms at the selective site of IIP. In the meantime, the concentration of H^+^ in the solution increases significantly under low pH, which results in the increase of the background current of the capacitance effect generated by differential pulse voltammetry. On the other hand, when the pH value is higher than 6, the metal ions may be hydrolyzed and the formation of negatively charged salts and hydroxyl complexes will prevent the binding sites of uranium to H_2_pdc in the polymer interaction, resulting in lower voltametric response. Therefore, MES buffer solution with pH = 6 was identified as the best condition for this experiment.

#### 3.3.4. Optimization of Enrichment Time

The influence of the enrichment time on the sensitivity of the uranyl sensor was investigated over a time period of 5–25 min while the condition of maintaining the other experimental conditions constant. It can be seen from Figure 5D that the corresponding voltametric signal strength increases with the increase of accumulation time up to 20 min and then remained almost constant. While, upon the further increase of the enrichment time of uranyl ions the intensity of the voltammogram signal remained basically unchanged and the enrichment time reached the adsorption equilibrium, which can be attributed to the adsorption saturation on the electrode surface. In order to shorten the analysis time as much as possible, improve the work efficiency and the sensitivity of the sensor, the time of 20 min was selected for further studies.

### 3.4. Study on the Performance of Uranyl Sensor

#### 3.4.1. Evaluation of Selectivity of MIIP/Fe_3_O_4_@SiO_2_/MCPE Sensor

**Figure 6:**
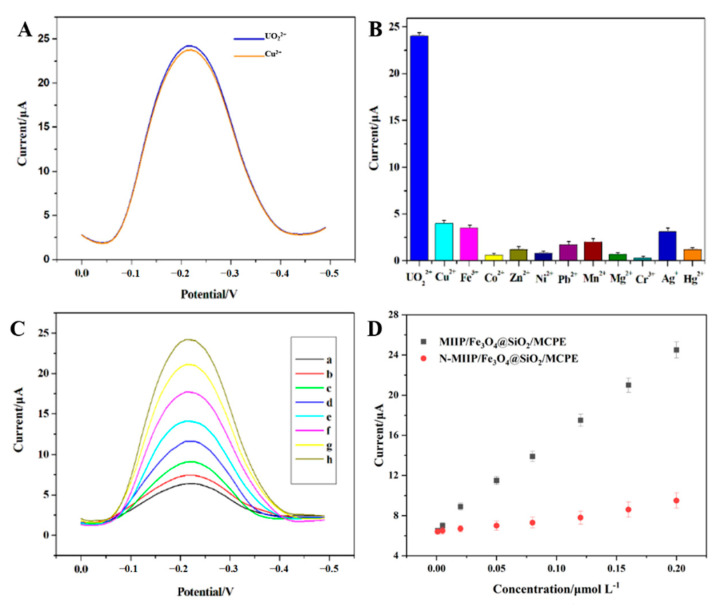
(**A**) The DPV signal of sensor after reaction in solution containing different metal ions; (**B**) when the Cu^2+^/UO_2_^2+^ ratio is 125:1, the influence of the presence of copper on the volt-ammetry signal of uranyl ions; (**C**) the MIIP/Fe_3_O_4_@SiO_2_/MCPE in different concentrations of UO_2_^2+^ (a: 0.001 μmol L^−1^; b: 0.005 μmol L^−1^; c: 0.02 μmol L^−1^; d: 0.05 μmol L^−1^; e: 0.08 μmol L^−1^; f: 0.12 μmol L^−1^; g: 0.16 μmol L^−1^; h: 0.2 μmol L^−1^) and (**D**) show the calibration curves of MIIP/Fe_3_O_4_@SiO_2_/MCPE.

The electrodes were incubated in solutions embodying the mixtures of UO_2_^2+^ (1.5 × 10^−7^ mol L^−1^) and various concentrations of some potentially interfering cations, followed by electrochemical analysis to investigate the selectivity of UO_2_^2+^ imprinted polymers in MIIP/Fe_3_O_4_@SiO_2_/MCPE (Figure 6B). Then, the solutions were a mixture of one metal ion and uranyl ion. It can be seen from Figure 6A that when the Cu^2+^/UO_2_^2+^ ratio is 125:1, the influence of the presence of copper on the voltammetry signal of uranyl ions is negligible. However, Cu^2+^ ions with a molar volume of more than 150 times can significantly affect the voltametric signal of UO_2_^2+^, indicating that these ions competed with each other when the concentration of interference ions is too high, which will occupy the selective holes in the IIP and affect the selective binding of UO_2_^2+^ on the upper cavity of the MIIP/Fe_3_O_4_@SiO_2_/MCPE. It is worth mentioning that the weakly adsorbed substance (the most common interference ion) has been removed from the electrode surface when the electrode is washed. Therefore, this washing process can also significantly reduce the interference effect to enhance the selectivity of the sensor. Within 95% confidence interval, the interference level of potential interference ions of uranyl ion of 1.5 × 10^−7^ mol L^−1^ are presented as following (the data is expressed as interference ion/UO_2_^2+^ ratio): Pb^2+^, Zn^2+^, Hg^2+^, Cd^2+^ (>150), Cu^2+^, Ag^+^, Mn^2+^, Fe^2+^ (>125), Cr^3+^, Co^2+^, Ni^2+^ (>175), NO_3_^-^, Cl^-^ (>150), PO_4_^3−^, CO_3_^2−^ (>200) and SO_4_^2−^ (>150). Obviously, these cation and anion sensors based on IIP exert no significant effect on the testing of UO_2_^2+^ ions by the IIP sensor.

#### 3.4.2. Sensor Stability and Repeatability

The repeatability of the sensor was verified for different concentration UO_2_^2+^ solutions by six continuous measurements using the same electrode MIIP/Fe_3_O_4_@SiO_2_/MCPE, and the relative standard deviation (RSD) was 1.53%, 2.15%, and 2.58%, respectively (Figure S7A). After storing the prepared sensors for 30 days, the electrochemical response signal was 95.08% of the original value, indicating the stability of the modified electrodes (Figure S7B). The results indicated that the repeatability and stability of the current response during the whole test were better.

#### 3.4.3. Calibration Curve and Detection Limit of Sensor

Under the optimal experimental conditions, a series of standard solutions of UO_2_^2+^ with different concentrations were manufactured. Differential pulse voltammetry (DPV) was employed to detect and calibrate the UO_2_^2+^ on the MIIP/Fe_3_O_4_@SiO_2_/MCPE at different concentrations, as shown in Figure 6C. The MIIP/Fe_3_O_4_@SiO_2_/MCPE sensor has a good linear relationship in the range of 1.0 × 10^−9^ mol L^−1^ to 2.0 × 10^−7^ mol L^−1^ with the detection and quantification limits of 1.08 × 10^−9^ and 3.23 × 10^−10^ mol L^−1^, respectively. The linear regression equation was expressed as I_P_(μA) = 86.77c(μmol L^−1^) + 7.218 with a correlation coefficient of r = 0.9993.

Dimovasilis et al. [52] developed a 6-O-palmitoyl-l-ascorbic acid (PAA)-modified graphite (GRA) electrode based on uranium concentration by non-homogeneous complexation followed by differential pulse voltammetry reduction for determination. As well, Ghoreishi et al. [53] performed the electrochemical determination of uranyl by using a Schiff base modified carbon paste electrode. Although the preparation process is simple, the detection range is limited and the detection limit is high. In addition, Shamsipur et al. [54] investigated the binding properties of uranyl ions to four different benzosubstituted macrocyclic diamides and prepared novel polymer film (PME) and coated graphite (CGE) UO_2_^2+^-selective electrodes. The CGE was used for flow injection potentiometry (FIP) for the determination of trace uranyl ions in samples. Although this potentiometric sensor exhibits the advantage of fast response and good selectivity, it is cumbersome to operate. Moreover, Metilda et al. [49] prepared a potentiometric ion-selective electrode (ISE) by dispersing uranyl ion-imprinted polymer particles in 2-nitrophenyl octyl ether (plasticizer) embedded in a polyvinyl chloride matrix. Its detection limit is low, but the electrode preparation method is complicated. Comparing the method of this experiment with other uranyl sensor electrochemical methods, which can be summarized from the comparison in Table 2 that the MIIP/Fe_3_O_4_@SiO_2_/MCPE sensor fabricated is more innovative and sensitive. This leads to the conclusion that the sensor designed with nanomaterials can greatly improve the limit of detection and linear range compared to other modified materials. In addition, the preparation procedure is simpler, cheaper, and more sensitive, thus the technique contributes significantly to the trace analysis of other substances.

### 3.5. Actual Sample Determination

The experimental data in Table 3 observed that the sample recoveries ranged from 96.29% to 103.22% with a relative standard deviation of 1.78% to 3.26%. Furthermore, it shows that the newly prepared MIIP/Fe_3_O_4_@SiO_2_/MCPE is suitable for the determination of trace uranyl ions in actual samples within a 95% confidence level and an acceptable range of error.

## 4. Conclusions

In this paper, a novel carbon paste electrode modified with core-shell structure Fe_3_O_4_@SiO_2_ ion imprinted membrane was constructed for the determination of trace uranyl ions in the heterogeneous environment. Prior to the preparation of imprinted polymers, density functional theory (DFT) was utilized to investigate the complex matrix interaction between templates and binding ligands so as to screen out the best functional binding ligands, which can also lay the theoretical basis for the rationally designing MIIP/Fe_3_O_4_@SiO_2_/MCPE. The simulation design not only simplifies the experimental process, saves cost and time, improves the modification efficiency, but also generates a stable structure and better performance of the synthesized polymers. The synergistic effect of Fe_3_O_4_@SiO_2_ modified IIP membranes on the modification of carbon paste electrode resulting in a larger specific surface area and excellent electron transfer capability on the electrode surface. Additionally, the magnetic core-shell imprinted film was obtained by embedding the magnet material inside the carbon paste electrode, allowing the polymeric microspheres to be simply immobilized to the electrode surface under the action of an applied magnetic field without the need for a complicated process. Moreover, it can solve the problem of interference caused by a series of electrochemical experiments on MIIP/Fe_3_O_4_@SiO_2_/MCPE in the background solution (such as template shedding) to minimize the impact, thereby rendering a stable and intact IIP structure on the electrode surface. Due to its unique recognition characteristics, the IIP-modified carbon paste electrode material is highly selective for the determination of uranyl in the presence of common interfering ions. To sum up, the sensor has the advantages of low cost, simple preparation, fast analysis, high sensitivity, good stability, and excellent reproducibility, which can be applied in real samples. In addition, the new sensor developed in the present study also lays a certain foundation for the determination of other metal ions.

## Figures and Tables

**Table 1:** Binding energy (∆E) of functional binding ligand complexes of different template molecules.

No	Complexes	Binding Energies (∆E/kJ mol^−1^)
1	1H-PPCA- UO_2_^2+^	−85.07
2	2-HP- UO_2_^2+^	−35.28
3	3-HP- UO_2_^2+^	−31.19
4	H_2_SA- UO_2_^2+^	−42.58
5	DHP- UO_2_^2+^	−49.13
6	3-PCA- UO_2_^2+^	−67.01
7	H_2_Pdc- UO_2_^2+^	−100.32

**Table 2:** Comparison of this method with other electrochemical methods for the determination of UO_2_^2+^.

Electrode	Method	Linear Range (mol L^−1^)	LOD (mol L^−1^)	References
Graphite electrodes	DPV	1 × 10^−8^–2.5 × 10^−7^	3.5 × 10^−8^	[52]
SHPMD/CNT/CPE	DPV	6 × 10^−7^–6 × 10^−8^	2 × 10^−9^	[53]
UO_2_^2+^−PME/CGE	FIP	1 × 10^−7^–1 × 10^−1^	5.4 × 10^−8^	[54]
UO_2_^2+^–DCQ–VP	ISE	2.0×10^-8^- 1.0×10^-2^	2.0×10^-8^	[49]
MIIP/Fe_3_O_4_@SiO_2_/MCPE	DPV	1 × 10^−9^–2 × 10^−7^	3.23 × 10^−10^	This work

**Table 3:** The MIIP/Fe_3_O_4_@SiO_2_/MCPE sensor applied to UO_2_^2+^ analysis and determination in actual samples (*n* = 6).

Sample	Added (μM)	Detected by This Method		
		Found (μM)	Recovery (%)	RSD (%)
Soil 1	0.10	0.103	103	1.69
Soil 2	0.10	0.970	97	2.01
Soil 3	0.10	0.101	101	2.41
Water 4	0.15	0.156	104	1.25
Water 5	0.15	0.147	98	3.06
Tap water	0.10	0.950	95	2.15
	0.15	0.146	97	1.03

## Data Availability

Not applicable.

## Supplementary Materials

The following supporting information can be downloaded at: https://www.mdpi.com/article/10.3390/s22124410/s1, Figure S1: Optimized conformation of 1H-PPCA, 2-HP, 3-HP, H2SA, DHP, H2Pdc, 3-PCA; Figure S2: The TEM images of (A) Fe_3_O_4_, (B) Fe_3_O_4_@SiO_2_; Figure S3: The EIS of (a) MIIP/MCPE, (b) MIIP/Fe_3_O_4_@SiO_2_/MCPE (after removal of the template), (c) MIIP/Fe_3_O_4_@SiO_2_/MCPE (after enrichment in 1.5 × 10^−7^ mol L^−1^ UO_2_^2+^ solution), (d) MIIP/Fe_3_O_4_@SiO_2_/MCPE (before elution of the template), (e) MCPE; (f) N-MIIP/ Fe_3_O_4_@SiO_2_/MCPE (impedance spectrum at 2 mmol L^−1^ K_3_Fe (CN)_6_/K_4_Fe (CN)_6_); Figure S4: The current response of the sensor was prepared with different crosslinking agents; Figure S5: The amount of crosslinking agent EGDMA (A), the amount of AIBN (B) and the reaction time of forming imprinted membrane (C); Figure S6: The effect of inorganic acid eluent on MIIP/Fe_3_O_4_@SiO_2_/MCPE determination of uranyl ions; Figure S7 (A) The effect of repeatability(a: 0.20 mol L^−1^, b: 0.15 mol L^−1^, c: 0.10 mol L^−1^ UO_2_^2+^ solution) on MIIP/Fe_3_O_4_@SiO_2_/MCPE; (B) Stability analysis of the sensing platform under 30 days with 0.15 μM target UO_2_^2+^ (orange), without target (green). The illustrated error bars represent the standard error of at least three independent measurements (*n* = 3).
