# Supplementary material for: A Novel Electrochemical Sensor Modified with a Computer-Simulative Magnetic Ion-Imprinted Membrane for Identification of Uranyl Ion
Source: Sensors (Basel). 2022 Jun 10;22(12):4410. doi: 10.3390/s22124410 (PMC9227270; doi:10.3390/s22124410)
Supplement: Supplementary file 1 [file sensors-22-04410-s001.zip › sensors-1737060-supplementary.pdf]

Electronic supporting information

A Novel Electrochemical Sensor Modified With a Computer-simulative Magnetic Ion-imprinted Membrane for Identification of Uranyl Ion

Li-Qiong He [1,†], Zhi-Mei Wang [2,†], Yu-Jie Li [2], Jing Yang [3], Li-Fu Liao [4], Xi-Lin Xiao [1,2,4,5*] and Yong Liu [2,*]

1 Hunan Key Laboratory of Typical Environmental Pollution and Health Hazards, School of Public Health, Hengyang Medical School, University of South China, Hengyang 421001, China; 20202014110972@stu.usc.edu.cn (L.-Q.H.)
2 School of Resource & Environment and Safety Engineering, University of South China, Hengyang 421001, China; 17872110128@163.com (Z.-M.W.); 20192002210069@stu.usc.edu.cn (Y.-J.L.)
3 Hengyang Market Supervision Inspection and Testing Center, Hengyang 421001, China; yjawb11@163.com (J.Y.)
4 School of Chemistry and Chemical Engineering , University of South China, Hengyang 421001, China; llfcxllc@163.com (L.-F.L.)
5 State Key Laboratory of Chemo & Biosensing and Chemometrics, Hunan University, Changsha 410082, China
* Correspondence: xiaoxl2001@163.com (X.-L.X.); 210000176367@usc.edu.cn (Y.L.)
† These authors contributed equally to this work.

Figure Captions

Figure S1 Optimized conformation of 1H-PPCA, 2-HP, 3-HP, H_2SA, DHP, H_2Pdc, 3-PCA.

Figure S2 The TEM images of (A) Fe_3O_4, (B) Fe_3O_4@SiO_2.

Figure S3 The EIS of (a) MIIP/MCPE, (b) MIIP/Fe_3O_4@SiO_2/MCPE (after removal of the template), (c) MIIP/Fe_3O_4@SiO_2/MCPE (after enrichment in 1.5×10^{-7} mol $L^{-1}UO_2^{2+}$ solution), (d) MIIP/ Fe_3O_4@SiO_2/MCPE (before elution of the template), (e)MCPE; (f)N-MIIP/Fe_3O_4@SiO_2/MCPE (impedance spectrum at 2 mmol L^{-1} K_3Fe $(CN)_6$/K_4Fe $(CN)_6$).

Figure S4 The current response of the sensor was prepared with different crosslinking agents.

Figure S5 The amount of crosslinking agent EGDMA (A), the amount of AIBN (B) and the reaction time of forming imprinted membrane (C) .

Figure S6 The effect of inorganic acid eluent on MIIP/Fe_3O_4@SiO_2/MCPE determination of uranyl ions

Figure S7 (A) The effect of repeatability(a: 0.20 mol L^{-1}, b: 0.15 mol L^{-1}, c: 0.10 mol L^{-1} UO_2^{2+} solution) on MIIP/Fe_3O_4@SiO_2/MCPE; (B) Stability analysis of the sensing platform under 30 days with 0.15 μM target UO_2^{2+} (orange), without target (green). The illustrated error bars represent the standard error of at least three independent measurements (n=3).

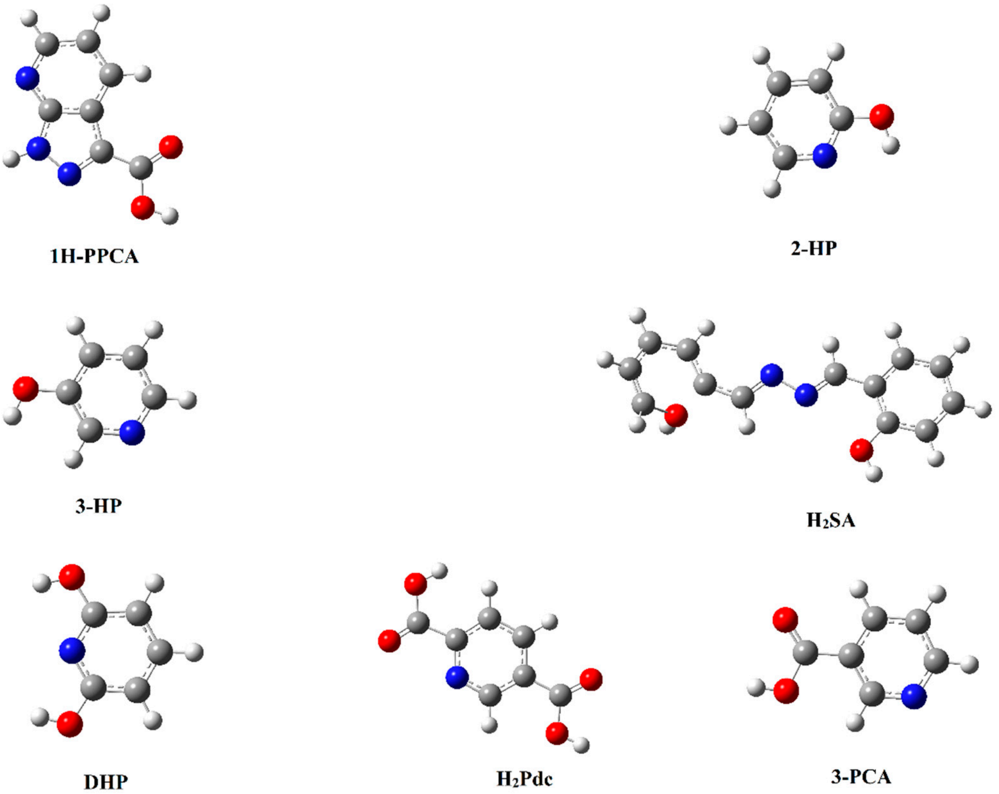

Figure S1 Optimized conformation of 1H-PPCA, 2-HP, 3-HP, H_2SA, DHP, H_2Pdc, 3-PCA.

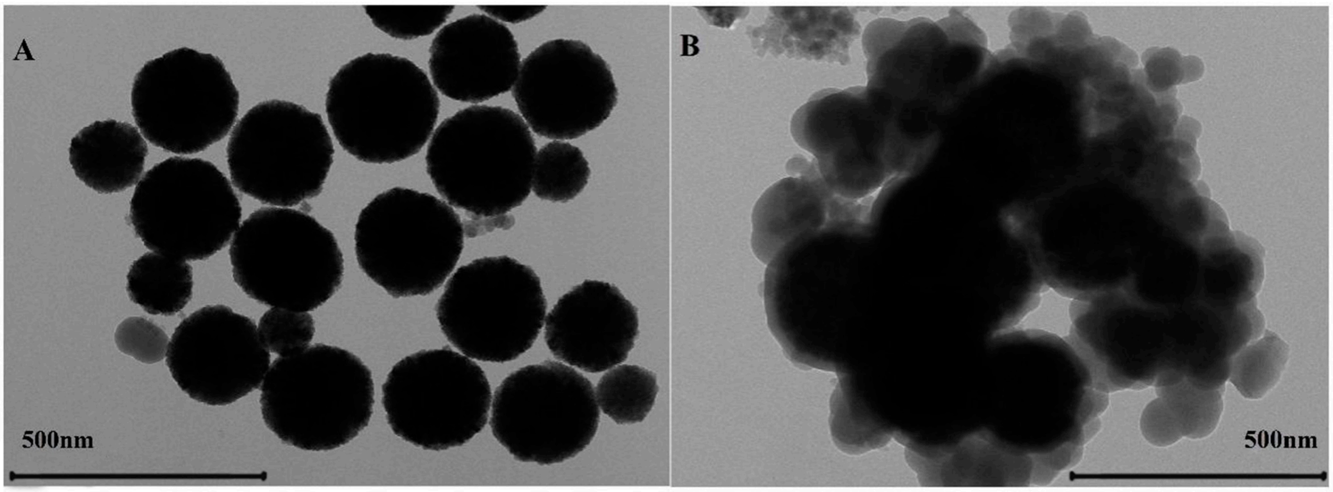

Figure S2 The TEM images of (A) Fe_3O_4, (B) $Fe_3O_4@SiO_2$.

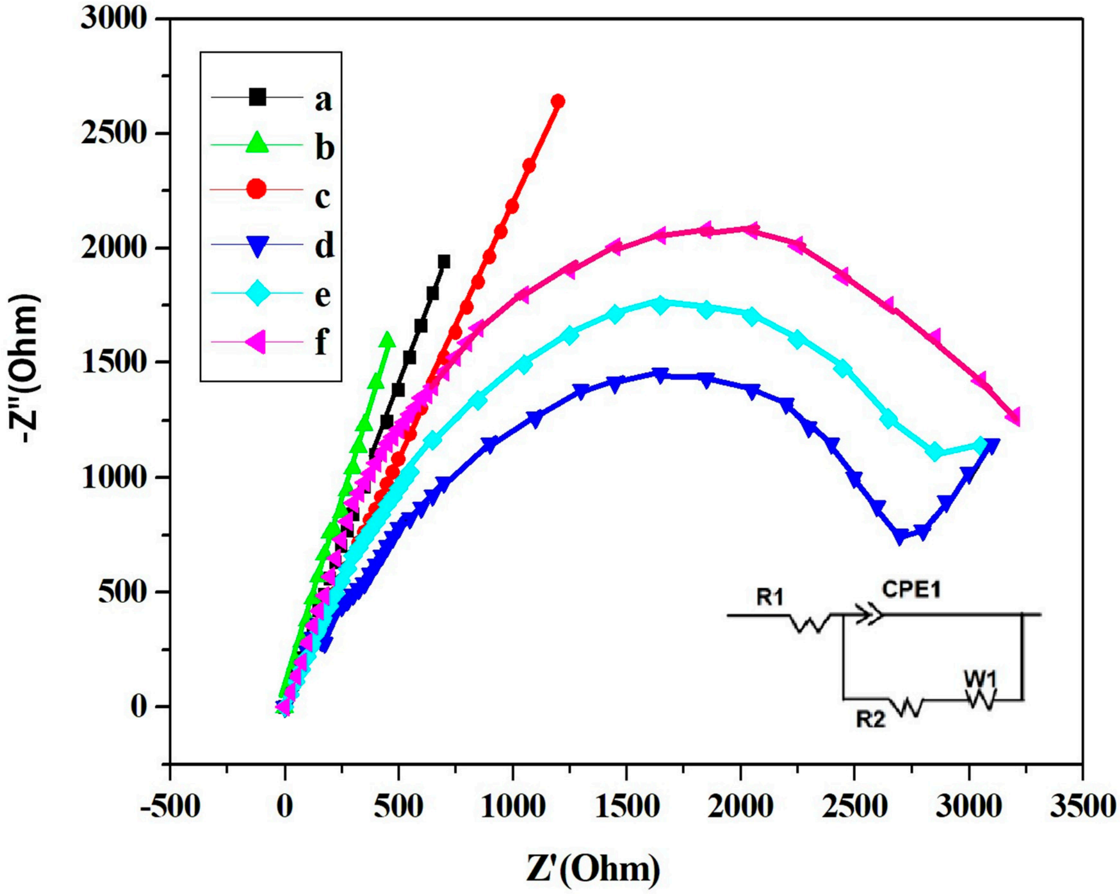

Figure S3 The EIS of (a) MIIP/MCPE, (b) MIIP/Fe_3O_4@SiO_2/MCPE (after enrichment in 1.5×10^{-7} mol L^{-1} UO_2^{2+} solution), (c) MIIP/Fe_3O_4@SiO_2/MCPE (after removal of the template), (d) MIIP/ Fe_3O_4@SiO_2/MCPE (before elution of the template), (e)MCPE; (f)N-MIIP/Fe_3O_4@SiO_2/MCPE (impedance spectrum at 2 mmol L^{-1} $K_3Fe\ (CN)_6$/$K_4Fe\ (CN)_6$).

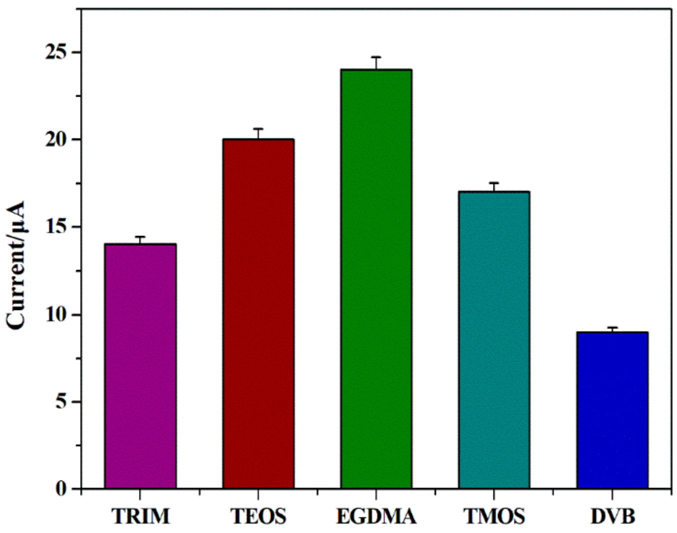

Figure S4 The current response of the sensor was prepared with different crosslinking agents.

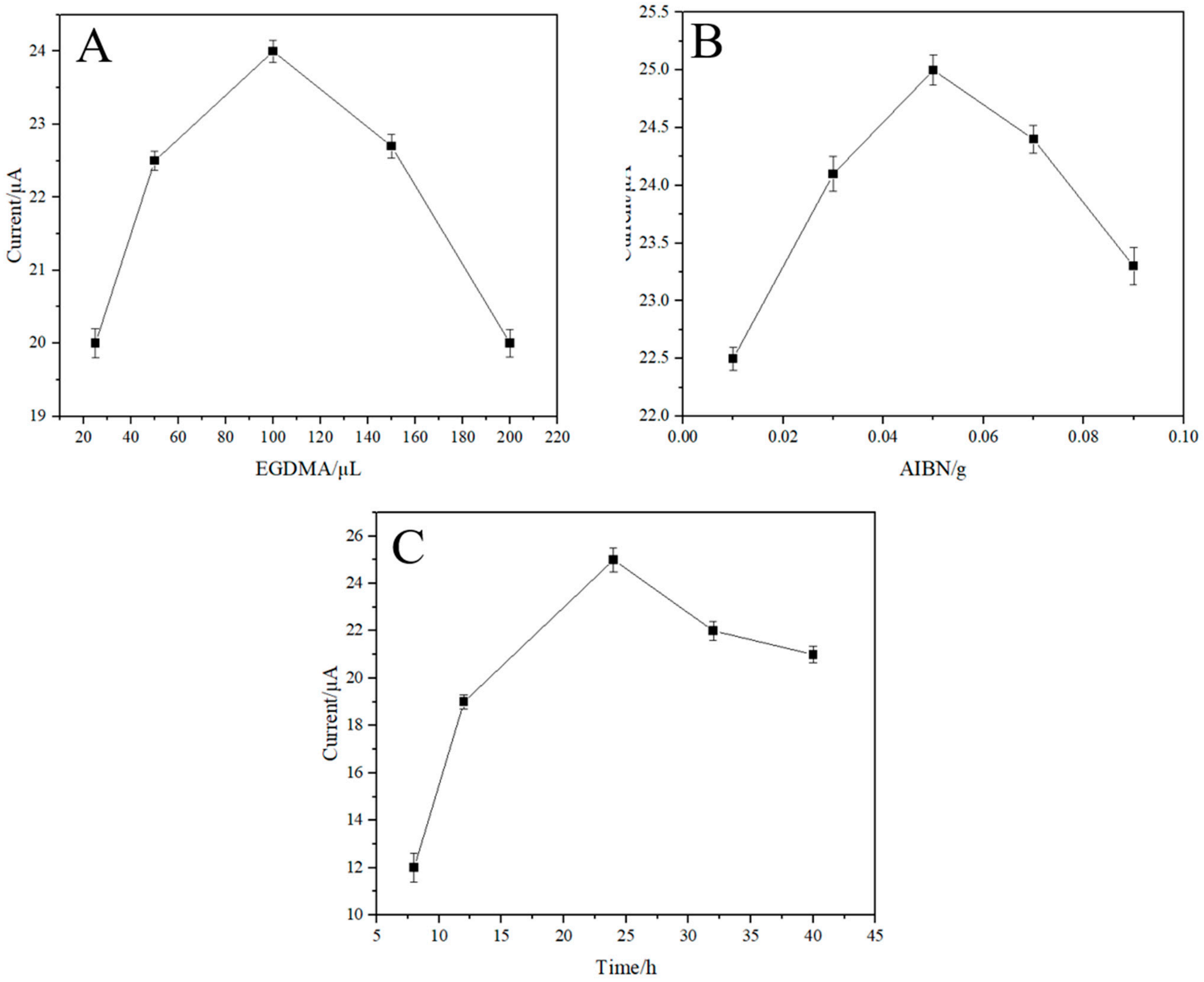

Figure S5 The amount of crosslinking agent EGDMA (A), the amount of AIBN (B) and the reaction time of forming imprinted membrane (C).

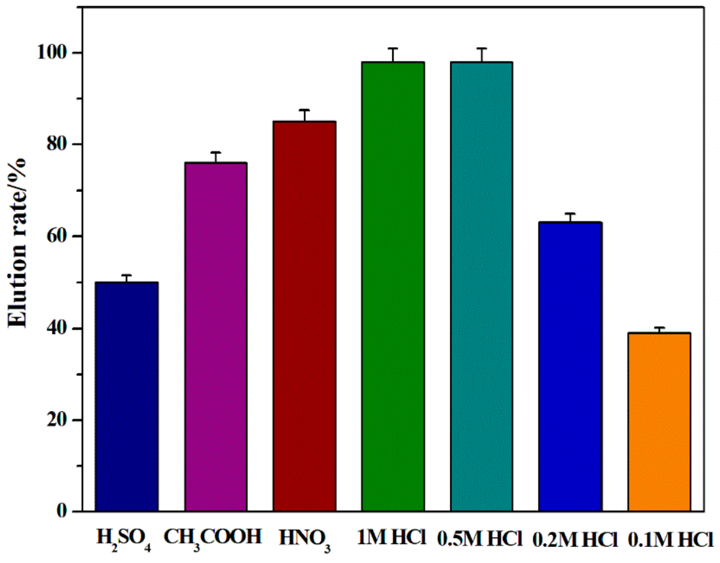

Figure S6 The effect of inorganic acid eluent on MIIP/Fe_3O_4@SiO_2/MCPE determination of uranyl ions.

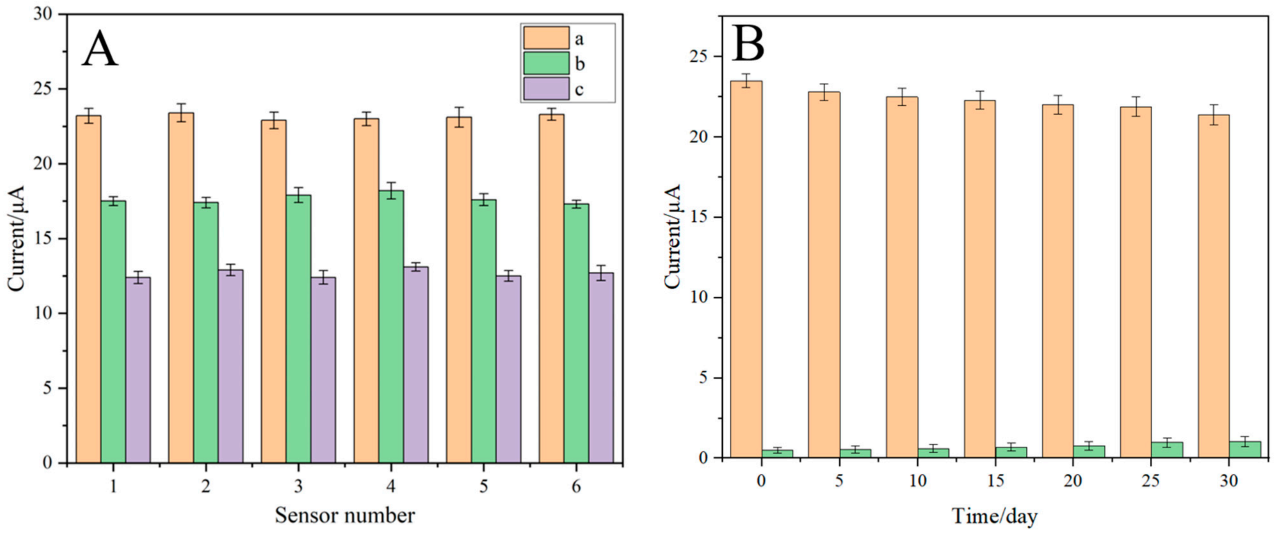

Figure S7 (A) The effect of repeatability(a: 0.20 mol L^{-1}, b: 0.15 mol L^{-1}, c: 0.10 mol L^{-1} UO_2^{2+} solution) on MIIP/Fe_3O_4@SiO_2/MCPE; (B) Stability analysis of the sensing platform under 30 days with 0.15 μM target UO_2^{2+} (orange), without target (green). The illustrated error bars represent the standard error of at least three independent measurements. (n=3)
